# Image and Speech Recognition Technology in the Development of an Elderly Care Robot: Practical Issues Review and Improvement Strategies

**DOI:** 10.3390/healthcare10112252

**Published:** 2022-11-10

**Authors:** Chin-Shyurng Fahn, Szu-Chieh Chen, Po-Yuan Wu, Tsung-Lan Chu, Cheng-Hung Li, Deng-Quan Hsu, Hsiu-Hung Wang, Hsiu-Min Tsai

**Affiliations:** 1 Department of Computer Science and Information Engineering, National Taiwan University of Science and Technology, Taipei 106335, Taiwan; 2 Administration Center of Quality Management Department, Chang Gung Medical Foundation, School of Nursing, Chang Gung University of Science and Technology, Taoyuan 33303, Taiwan; 3 College of Nursing, Kaohsiung Medical University, Kaohsiung 807378, Taiwan

**Keywords:** elderly care robot, long short-term memory, convolutional neural network, gaze target fixation point, continuous bag of words, pose recognition, facial expression recognition, eye gazing recognition, speech recognition

## Abstract

As the world’s population is aging and there is a shortage of sufficient caring manpower, the development of intelligent care robots is a feasible solution. At present, plenty of care robots have been developed, but humanized care robots that can suitably respond to the individual behaviors of elderly people, such as pose, expression, gaze, and speech are generally lacking. To achieve the interaction, the main objectives of this study are: (1) conducting a literature review and analyzing the status quo on the following four core tasks of image and speech recognition technology: human pose recognition, human facial expression recognition, eye gazing recognition, and Chinese speech recognition; (2) proposing improvement strategies for these tasks based on the results of the literature review. The results of the study on these improvement strategies will provide the basis for using human facial expression robots in elderly care.

## 1. Introduction

Many developed countries are currently faced with issues brought about by changing population structures as societies age. These issues include significant increases of life expectancy and elderly populations. Statistical data of the global population in 2019 indicated that the average global life expectancy was 72 years in 2019, with 74.2 years for women and 69.8 years for men [1]. The average life expectancy in Taiwan in 2019 was 80.9 years, with 77.7 years for men and 84.2 years for women. The National Development Council predict that Taiwan will become a super-aged society by the year 2026, by which time the elderly populations in Taiwan will account for 20.5% of the total population, and 41.2% by the year 2065 [2]. As the elderly population increases dramatically, the issue of elderly care has become a pressing public health issue for the world.

As the world’s population is aging and there is shortage of sufficient caring manpower, the development of intelligent care robots is a feasible solution. The development of care robotics around the world has primarily been focused on assistive devices, as well as on providing assistance in social interaction. Examples of these robots include FujiSoft’s PALRO care robot from Japan, which focuses on care support; the NAO robot by France’s Aldebaran, which is a humanoid robot focusing on interaction; the Kirobo Mini, which is a small companion robot developed by Toyota; the PARO developed by Japan’s National Institute of Advanced Industrial Science and Technology, which is a seal-type robot utilized in the care of older people with dementia. Additionally, Pepper is a multi-functional robot capable of emotional expressions, which is produced by Taiwan’s Hon Hai Precision Industry, and Zenbo is a household companion robot developed by Taiwan’s ASUS Corporation. Although a plenty of care robots have been developed, humanized care robots that can suitably respond to the individual behaviors of elderly people, such as pose, expression, gaze, and speech, are generally lacking. In order to implement a humanized care robot, this research has two main objectives: (1) to conduct a literature review and status analysis of the four core tasks of image and speech recognition technology: human pose recognition, face detection, eye gazing recognition and Chinese speech recognition; (2) to propose improved strategies for these tasks. The discovery of these improved strategies will provide the basis for the use of expressive robots in elderly care.

## 2. Literature Review

This study attempts to realize four core tasks with image and speech recognition technology: human pose recognition, human facial expression recognition, eye gazing recognition, and Chinese speech recognition. These tasks will lay the foundation for creating the functionality of expressive robots in elderly care. What follows is a review of the literature on these four tasks.

### 2.1. Review of Human Pose Recognition

Body gesture is one of the fundamental skills of a person. The postures of the human body are always changing in daily life, each one representing the appearance state of a person. Real-time interpretation of the significance behind each state could lead to many applications, such as personal safety and behavior prediction. Thus, making human pose recognition is a popular subject of investigation.

Traditional methods of image recognition include the least-square method, a globally convergent iterative method, and simplified linear solutions [3], which already have a certain level of precision. The advent of machine learning has effectively improved the prediction accuracy of image recognition models. Machine-learning-based recognition is usually through supervised learning, in which the recognition model learns the features of datasets using labeled data, continuously adjusts the basis of interpretation, and is trained to predict unknown classifications of input data via a set of decision criteria. This technology further enhances the recognition capability of the care robot. The common machine-learning-based recognition methods include support vector machine (SVM) [4] and random decision forest [5].

**Figure 1:**
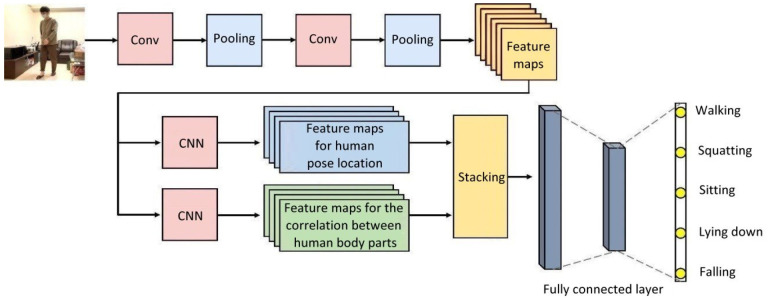
Common CNN architecture used in human pose image recognition.

In recent years, deep-learning architectures such as the convolutional neural network (CNN) and long short-term memory (LSTM) have paid wider attention to, and have been utilized in, developing the recognition capabilities of care robots. Figure 1 demonstrates the steps and procedures of using CNN architecture [6] in the image recognition of human poses, which is provided as a reference for future research and development.

### 2.2. Review of Human Facial Expression Recognition

Human facial expressions are key to social interaction and form the basis of non-verbal human communication. Taking elderly care as an example, one must learn to communicate and interact with elderly people through eye contact, expressions, and body language. Traditionally, there are two widespread methods for classifying human facial expressions. The first method is based on the Bayesian classifier, which classifies an unknown facial expression based on known data of human facial expressions. For example, the hidden Markov model (HMM) [7] evolves from the Bayesian classifier. The second method is classification based on distance metrics, such as the support vector machine (SVM) [8], which is a supervised learning algorithm that uses linear regression to analyze data. This method has good recognition accuracy and can better classify human facial expressions.

**Figure 2:**
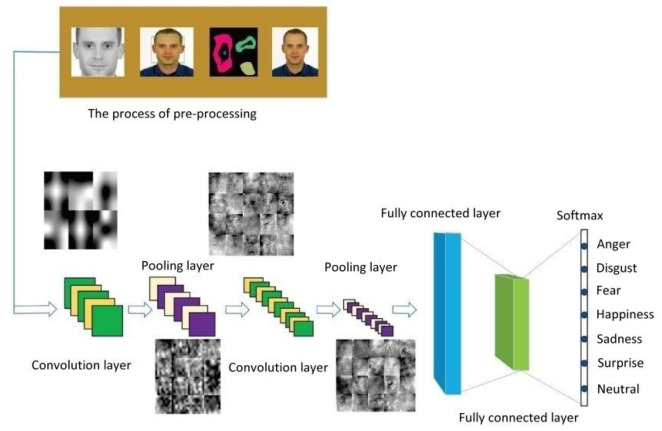
CNN architecture of human facial expression image recognition.

Our study will adopt a convolutional neural network (CNN) as our deep learning model [9]. The architecture of this model will be used to recognize seven kinds of elderly facial expressions: anger, disgust, fear, happiness, sadness, surprise, and neutral. Figure 2 shows a CNN architecture that can be used by a care robot to recognize human facial expressions. Because multiple functional modules will be loaded into the computer equipped in the care robot, we will propose an improved CNN architecture for facial expression recognition, where the convolutional model is streamlined to reduce the computational loading. In addition, we focus on suitable convolutional kernel quantity design to raise the accuracy of elderly facial expression recognition.

### 2.3. Review of Eye Gazing Image Recognition

Eyes are so called the “window to the soul” and are the primary means for understanding how information is obtained through the gaze. Recently, eye tracking technology has been widely researched to better analyze the target fixation point for eye gazing.

The development of eye tracking has gone through numerous advances. Rapid advancements in computer graphics have promoted the development of highly sophisticated eye tracking systems, and greatly enhanced the application of eye tracking research in psychology and related fields. Therefore, the study on eye tracking has become a benchmark of modern psychology research.

**Figure 3:**
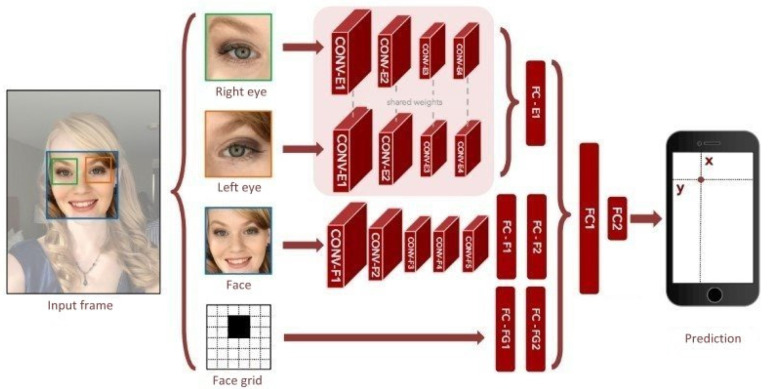
CNN architecture of eye gazing image recognition.

Eye gazing image recognition technology serves as capturing the images of eye movements and converting them to image coordinates, including the pupil positions and fixation points [10]. Essentially, this technology “decodes” the captured images of eye movements and converts them for use in detecting and tracking the dynamic vision of the elderly. Figure 3 shows the CNN architecture frequently employed in eye gazing image recognition. Common eye gazing image recognition models are usually combinations of several convolutional layers and fully connected layers [11]. Such technology first locates a human face, then individually obtains right/left eye parts, human face part, and facial features, and feeds them into the neural network to generate a feature map. This feature map is then passed through a spatial weighting scheme to generate a weight graph, which is then multiplied with the feature map using matrix multiplication. Finally, the generated map is inputted into the fully connected layer to predict the coordinates of the target fixation point for eye gazing, achieving the effect of tracking elderly eyes by the care robot.

### 2.4. Review of Chinese Speech Recognition

Speech recognition is the ability of a computer to receive and process vocally spoken commands, which is known as automatic speech recognition (ASR) [12]. After converting “speech to text” (STT), the Jieba is used to complete segmentation of Chinese words. This package generates a directed acyclic graph (DAG) for all possible word combinations and uses dynamic programming to query the probable segmentation combination based on word frequency; for unknown words, the hidden Markov model (HMM) for Chinese words and the Viterbi algorithm [13] are used to find the possible words with the highest degree of similarity.

**Figure 4:**
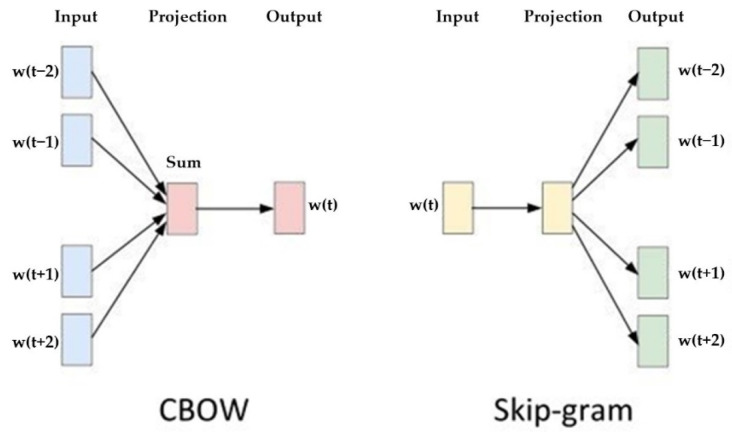
Two most popular frameworks of word2vec: (**left**) CBOW; (**right**) skip-gram [15].

After decades of development, speech recognition technology has been infused with other word processing technology. This technology is referred to as natural language processing (NLP) [14]. In the past, the majority of text representation in NLP was through the use of one-hot encoding. Although this method is easy to understand and calculate, it nevertheless has some limitations, such as an inability to consider the sequence order between words, and assuming words are independent from each other. This was until Mikolov et al. proposed a new word recognition model framework called “word2vec” in 2013 [15], which has been used to improve the NLP technology. As indicated in Figure 4, it has two main models: continuous bag of words (CBOW) and skip-gram. By employing one of these language processing models on the Chinese speech processing of a care robot, we can project the words in a higher dimensional space and calculate the cosine similarity to predictive results of the surrounding context.

The technology that converts the predicted answers to spoken voice is called “text to speech” (TTS) technology. Conventionally, text-to-speech can be realized via two methods: concatenative speech synthesis and parametric speech synthesis [16]. Currently, the WaveNet model proposed by the Google DeepMind team in 2016 has received widespread attention [17]. WaveNet synthesizes sound signals by using a neural network method trained with recordings of real speech. Therefore, through this voice synthesis technology, robot speech can become more natural and closer to real speech.

## 3. Improvement Strategies

In this study, researchers played the role of testers to propose the most optimal solutions for the development of care robots, drawing from the results of the literature review of image and speech recognition technology to fulfill the four core research tasks. Aside from using the traditional method for gazing tracking, all other tasks will be achieved through deep-learning techniques to conduct human pose recognition, facial expression recognition, and Chinese speech recognition. The systemic processes, frameworks, algorithms, and outcome examples of each respective technique will be described in order below.

### 3.1. Methods for Human Pose Image Recognition

**Figure 5:**
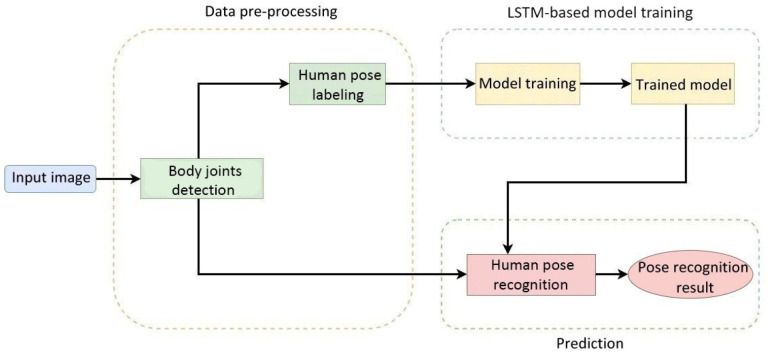
Flowchart of the human pose image recognition system.

According to statistics, the probability of accidental falls occurring increases with age. Elderly people falling has become the third major cause of chronic disability for elderly people. Therefore, we propose a process of the human pose image recognition system used in care robots, which is shown in Figure 5. This procedure comprised three parts: the pre-processing of elderly body images required for model training, the model training phase, and the model testing phase.

### 3.2. Pre-Processing of Data

**Figure 6:**
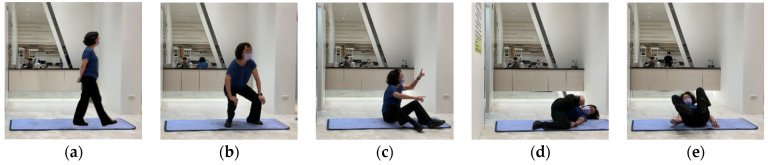
Five kinds of human pose images in a fall event detected: (**a**) walking; (**b**) squatting; (**c**) sitting; (**d**) lying down; (**e**) falling.

The purpose of pre-processing elderly images is to train the human pose recognition model used in care robots. First, a camera recorded the movements of elderly people. Then, we used a toolkit to detect the positions of the key joints of the human body in 3D space. The data were then converted to a vector for training. Different from most datasets of pose recognition, this research collected a sequence of movements that occur within 30 frames of a video as one training data input. Lastly, the data were labeled with five common poses of the elderly, as shown in Figure 6. These five kinds of human poses act as the basis of feedback processing by care robots for the recognition of falls.

### 3.3. Model Training Phase

**Figure 7:**
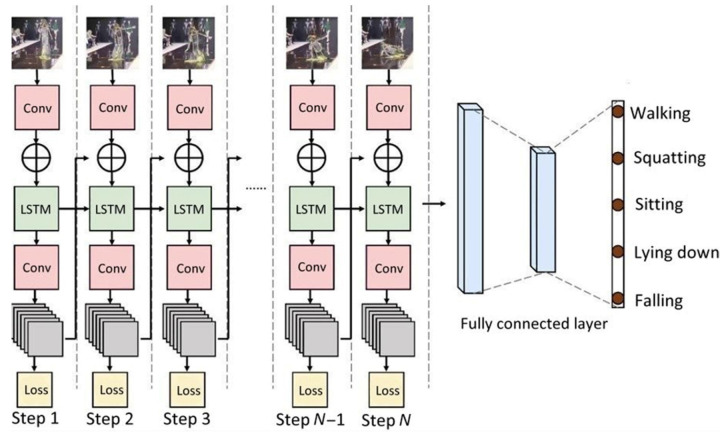
LSTM-based architecture of the elderly fall detection model.

The deep-learning architecture used in this study utilized three layers of LSTM [18]. In the training phase, 5-fold cross validation was used to decrease overfitting or bias and increase the generalization of the model. Figure 7 shows the system architecture of the LSTM-based fall recognition model.

### 3.4. Methods for Human Facial Expression Recognition

**Figure 8:**
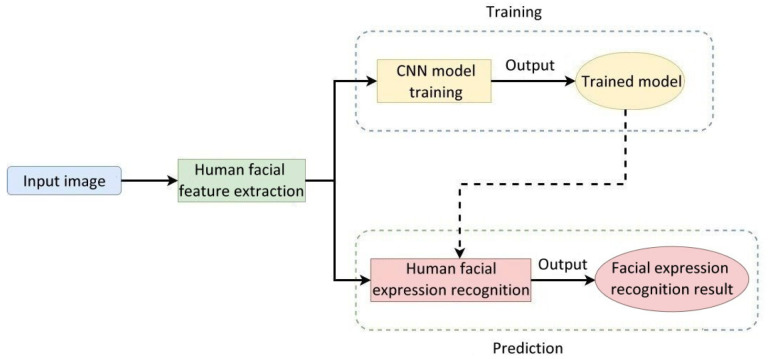
Flowchart of the human facial expression recognition system used in care robots.

Figure 8 illustrates the flowchart of the facial image recognition system used in care robots. Human facial features were extracted from the input human face images and imported into the CNN for training. During the recognition phase, the trained model can recognize human facial expressions from the input features of a human face.

There are three major procedures in the recognition of human facial expression images, which are described as follows:

1. Extracting Haar-like features from a human face image

Haar-like features are digital image features used in object recognition. Its name is originated from its similarity to the Haar wavelet, and it was the first method that could extract human facial features in real time [19]. In this study, the outline of a human face can be acquired from every frame of the images captured by the camera of a care robot.

2. Recognizing facial expression images by a convolutional neural network

**Figure 9:**
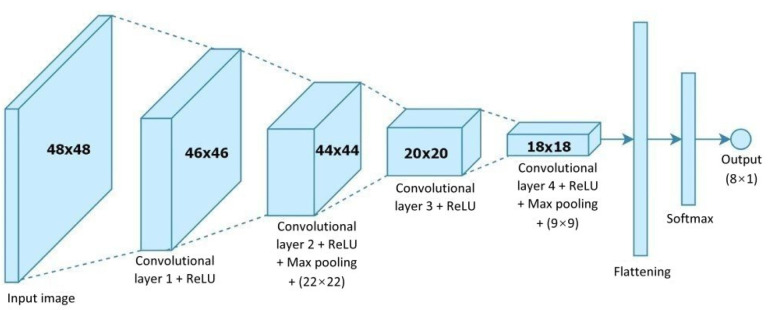
CNN architecture of human facial expression recognition.

**Figure 10:**
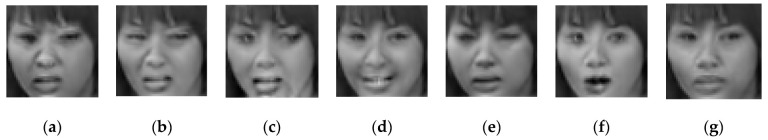
Seven kinds of facial expressions: (**a**) anger; (**b**) disgust; (**c**) fear; (**d**) happiness; (**e**) sadness; (**f**) surprise; (**g**) neutral.

Figure 9 shows a CNN architecture for use in human facial expression recognition, which mainly consists of four convolutional layers. The size of the face image fed to this architecture is 48 × 48 pixels and is passed through four 3 × 3 kernels of convolutional computation. The resulting image then enters the flattening stage after passing through the 4th convolutional layer, and then finally through the normalized exponential function (softmax) layer to obtain the kind of facial expression whose value is the maximum of the seven. Figure 10 shows an example of seven kinds of facial expressions.

3. Locating the human face and displaying the facial expression recognition result

**Figure 11:**
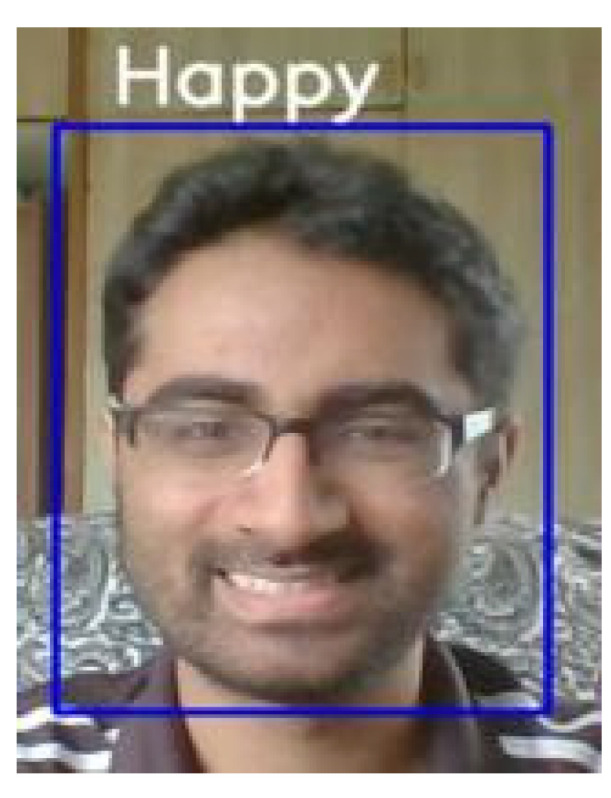
An example of facial expression recognition by our CNN model.

Figure 11 shows an actual example of facial expression recognition in this study. Because the scale of the neural network model will affect the recognition performance, we set out a trade-off between architecture and accuracy to elicit optimal performance. As a result, our network model would not be overly complicated and would have good recognition accuracy, as well as allowing real-time computation on low-cost hardware.

### 3.5. Methods for Eye Gazing Image Recognition

**Figure 12:**
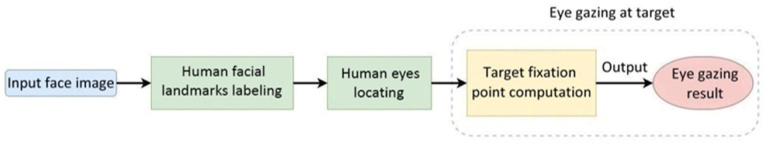
Flowchart of the eye gazing image recognition system used in care robots.

Figure 12 shows the flowchart of the eye gazing image recognition system used in care robots. After locating human eyes from the facial landmarks, the target fixation point was then computed to output the recognition result of eye gazing.

**Figure 13:**
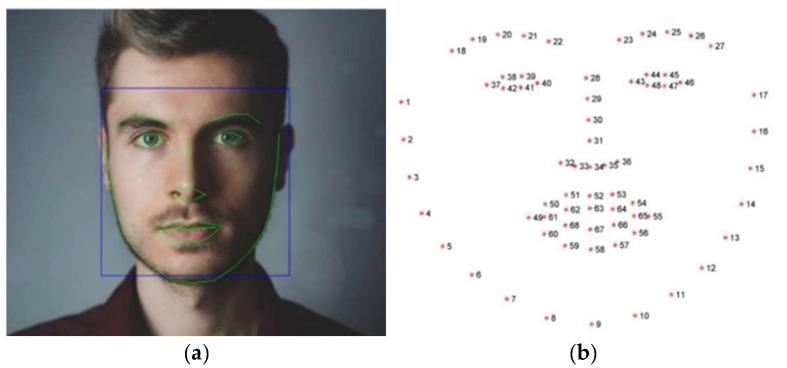
Illustration of 68 landmarks in a human face image: (**a**) landmark locations in green color; (**b**) landmark numbers.

First, we employed the C++ library called Dlib to detect human faces of an input image. For a detected human face, 68 landmarks were then labeled using a dynamic optical flow approach [20] as shown in Figure 13.

**Figure 14:**
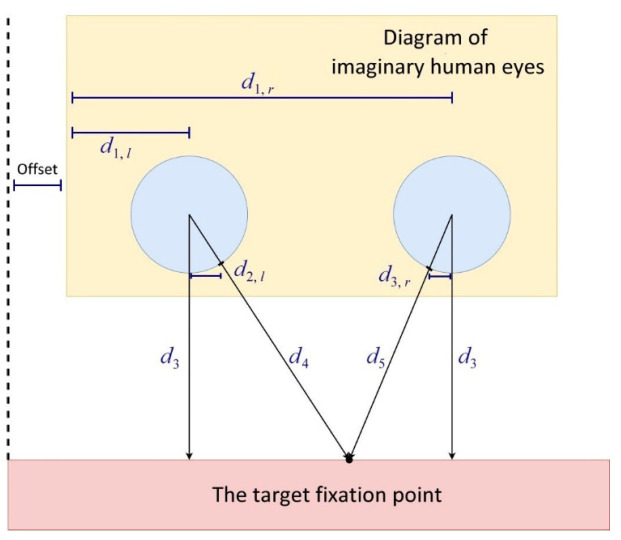
Geometry relationship of the target fixation point for eye gazing.

According to the intercept theorem, one can calculate the horizontal coordinates from the center of an eye to the target fixation point for gazing. The horizontal offset of the camera lens can be compensated with a constant. Figure 14 illustrates the geometry relationship of calculating the coordinates of a target fixation point for eye gazing.

Taking the left eye as an example, Equation (1) is used to calculate the coordinates of the gaze target fixation point on the *x* axis:

(1) $$d_{l}=\alpha_{l}\cdot d_{1,l}+\beta_{l}\cdot d_{2,l}+\gamma_{l}$$

Here, $d_{1,l}$ is the position of the center of the left eye on the x axis; $d_{2,l}$ is the distance between the center of the left eye and where the rim of the left eye intersects with the left gaze line, and an image calibration process is required to obtain the coefficients $\;\alpha_{l}$, $\beta_{l}$, and $\;\gamma_{l}$. Similarly, by using the position of the center of the right eye on the x axis ($d_{1,r}$) and the distance between the center of right eye and where the rim of the right eye intersects with the right gaze line ($d_{2,r}$)—as expressed in Equation (1)—we can again calculate the coordinates of the gaze target fixation point on the x axis ($d_{r}$).

### 3.6. Methods for Chinese Speech Recognition

**Figure 15:**
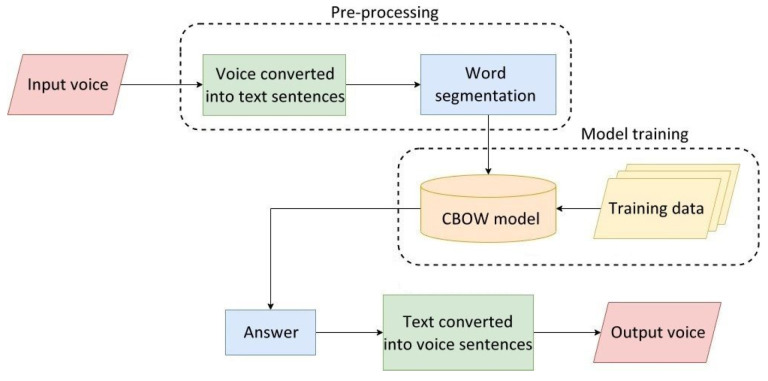
Flowchart of the Chinese speech recognition system used in care robots.

Figure 15 shows the flowchart of the Chinese speech recognition system used in care robots, whose three main procedures are described as follows:

#### 3.6.1. Hidden Markov Model (HMM) and Viterbi Algorithm

After receiving the speech of elderly people and generating text, the Chinese words were segmented with Jieba, which utilizes the HMM and Viterbi algorithm. From Equations (2) and (3), *S* is the state space for the HMM. Assuming there are *k* states, and the initial state is *I*, then $\pi_{i}$ is the probability of the initial state, $\pi_{i,j}$ is the probability of state *i* transitioning to state *j*, $y_{1}\sim y_{t}$ are the observed outputs, and $x_{1}\sim x_{t}$ are the most probable output states observed.

(2) $$V_{1,k}=P\left(\left.y_{1}\right|k\right)\cdot \pi_{k}$$

(3) $$V_{t,k}=max_{x\epsilon S}\left(P\left(\left.y_{t}\right|k\right)\cdot \pi_{x,k}\cdot V_{t-1,x}\right)$$

where $V_{t,k}$ is the probability of the most probable state sequence $y_{1}\sim y_{t}$ responsible for the previous *t* observations with *k* as the final state. Additionally, $P\left(\left.y_{t}\right|k\right)$ is the probability of the generated output $y_{t}$ that corresponds to state *k*.

In this study, the HMM and Viterbi algorithm were utilized to compute the text of elderly Chinese speech and output the most possible word sequence via Equation (4), so as to obtain the final results of Chinese word segmentation. When t>1 and computing the optimal corresponding state sequence, the function $argmax\left(\cdot \right)$ was used to compute the most probable observed output state $x_{t}$ and the function $Ptr\left(\cdot ,\;t\right)$ returns the optimal Viterbi path from the second-to-last equation.

(4) $$x_{t}=argmax_{x\epsilon S}\left(V_{t,\;x}\right)\;x_{t-1}=Ptr\left(x_{t},\;t\right)$$

#### 3.6.2. CBOW Architecture

**Figure 16:**
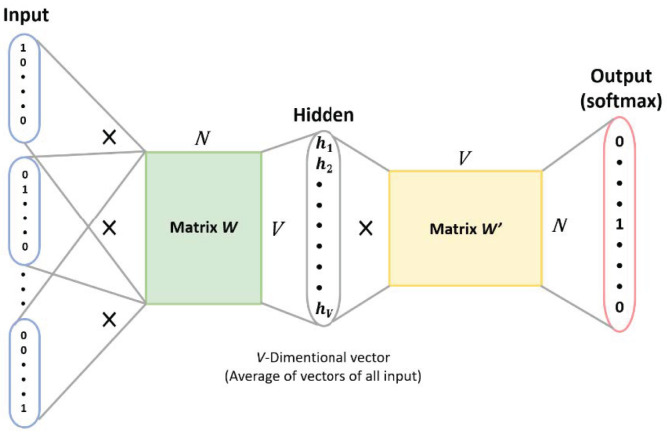
CBOW architecture used in Chinese speech recognition [21].

We used the CBOW model to identify speech meaning in the elderly. The CBOW is a neural network as shown in Figure 16, which consists of the input layer for receiving one-hot encoded tensors as network input. The hidden layer is formed by multiplying the tensors with the tensors of the word embedding to obtain the tensors of the context words. Additionally, the output layer multiplies the results of the hidden layer with the tensor of another word embedding, which is then fed into the softmax layer to obtain the prediction results of the context words in relation to the middle word, allowing the care robots to understand elderly speech.

#### 3.6.3. gTTS Speech Synthesis Module

The gTTS library was employed to convert text to speech, so the care robot can respond with speech. Essentially, gTTS adopts Google text-to-speech API, which uses WaveNet to generate a speaking voice. The core architecture of WaveNet is the dilated causal convolution, which is a deep learning model to generate raw audio. Such a model performs that the string replies of a care robot serve as the input to carry out speech synthesis. It can emit human-like voice to make inanimate robots behave more humanly.

## 4. Test Results and Analysis

After multiple field tests and verification, this study proposes optimal solutions for the four core tasks of the care robot. The following sections evaluate the test results of human pose recognition, facial expression recognition, eye gazing recognition for live videos, as well as those of the Chinese speech recognition for live audios.

### 4.1. Human Pose Image Recognition Test

**Figure 17:**
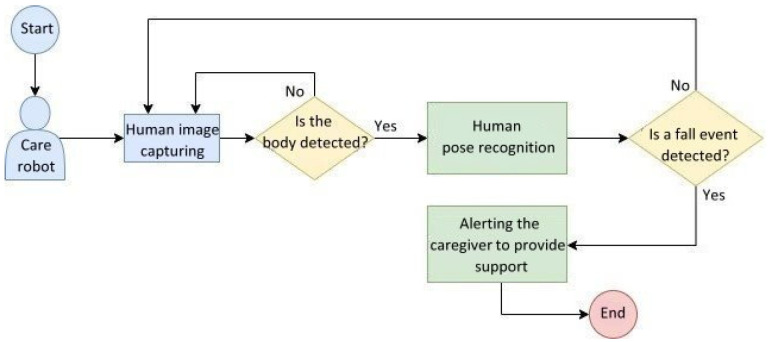
Behavioral flowchart of the human pose recognition system embedded in care robots.

To simulate the human pose recognition test scenario for elderly people, we illustrate a behavioral flowchart of the care robot encountering the elderly as shown in Figure 17. First, the system obtains the human pose image of elderly people from a camera and detects whether the image contains a human pose of the elderly. If not, the system continues to capture images; otherwise, the system then recognizes the elderly body poses. If a fall event is detected, the system alerts the caregiver to provide support; otherwise the system continues to capture images from the camera for recognition. This flowchart outlines a concept for future developers to realize the human pose recognition system embedded in care robots. The steps include human images captured from a camera, the human pose recognition module, and system feedback in the case of a fall event being detected.

**Figure 18:**
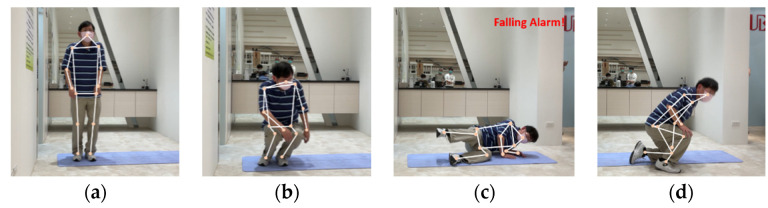
Representative recognition results from the video of a falling elderly man: (**a**) Frame 218 (walking); (**b**) Frame 376 (squatting); (**c**) Frame 423 (falling); (**d**) Frame 823 (squatting).

In this study, we adopted the LSTM architecture to implement the human pose recognition module. It not only achieves good recognition accuracy, but it also effectively improves the lack of continuity between frames and the costly operation of a CNN-based human pose recognition model. To evaluate the efficacy of the care robot for elderly people, we filmed many videos that took place in an indoor environment and verified the accuracy of the falling recognition. The care robot only considers the largest target (an elderly person) in the lens. Figure 18 shows the representative test results of real-time pose recognition from the video (length: 134 s) of a falling elderly man. The annotation of the falling alarm is displayed in the upper right corner of the monitor equipped in a care robot.

For the convenience of evaluation, each 30-frame shot of a video acts as a single pose datum. Table 1 records the confusion matrix for the pose recognition results of 168 shots from the video of the falling elderly man. Table 2 lists the precision, recall, and accuracy rates as well as the F1-score according to the confusion matrix. This outcome indicates that since falling is composed of continuous movements, it has more variations and is thus relatively harder to predict.

### 4.2. Human Facial Expression Recognition Test

**Figure 19:**
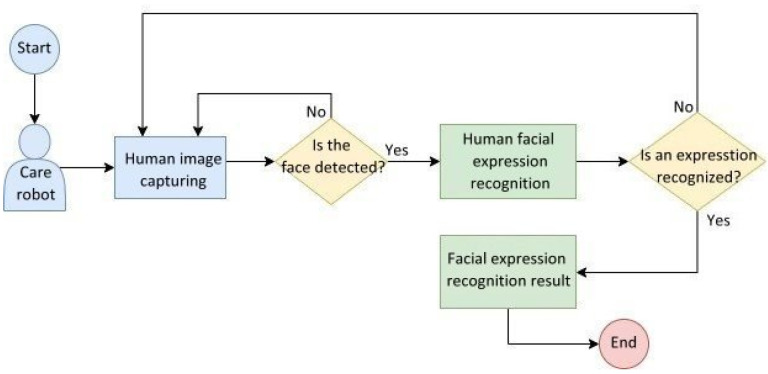
Behavioral flowchart of the human facial expression recognition system embedded in care robots.

Figure 19 illustrates the behavioral flowchart of the care robot whose embedded system captures a human image from the camera and detects in real time whether a human face appears in the image. If yes, the facial expression is then recognized; if not, the camera continues to capture images. Then, the facial expression recognition is accomplished and outputted to display; otherwise, the camera continues capturing images.

**Figure 20:**
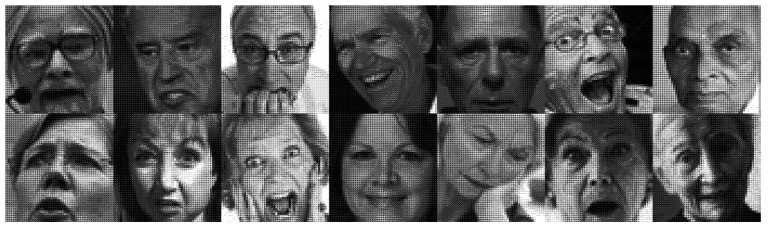
Sample images of human facial expressions of elderly man and woman in the FER-2013 dataset.

Over the course of developing the human facial expression recognition model used in the care robot, we employed the FER-2013 dataset for training and verification. Figure 20 shows some sample images of human facial expressions in the FER-2013 dataset.

During the process of model training, we evaluated the convergence of the model through the loss function of the categorial cross entropy as expressed in Equation (5).

(5) $$Cross\;entropy\;Loss=-\overset{C}{\underset{c=1}{\sum }}\overset{n}{\underset{i=1}{\sum }}y_{c,i}log_{2}\left(p_{c,i}\right)$$

where C is the total number of categories of the human facial expressions, n is the total number of human facial expression images, $y_{c,i}$ indicates the binary indicator (0 or 1) from one hot encode (the ith human facial expression that belongs to the cth category of real human facial expression), and $p_{c,i}$ indicates the probability of predicting the ith human facial expression image belonging to the cth category.

In this study, we randomly sampled 100 images from the aforementioned dataset. Table 3 shows the statistics of the recognition of the seven kinds of human facial expression images, represented as a confusion matrix. Table 4 is the evaluation data of the above recognition result.

### 4.3. Eye Gazing Recognition Test

**Figure 21:**
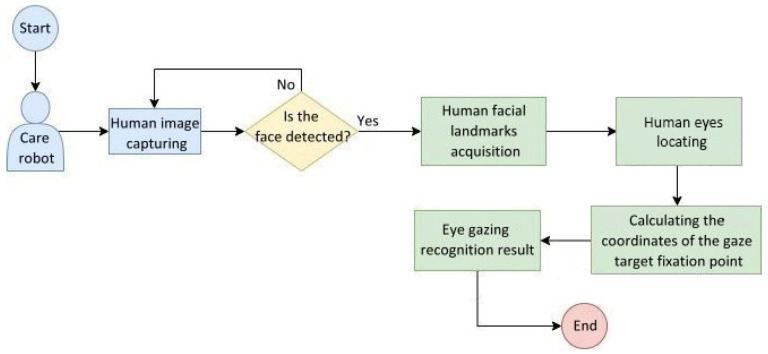
Behavioral flowchart of the eye gazing recognition system embedded in care robots.

Figure 21 shows the behavior flowchart of the eye gazing image recognition system embedded in the care robot. The system captures a human image from the camera and detects the presence of a human face in real time. If one is not detected, the camera continues to capture images; otherwise, the facial landmarks are acquired to locate human eyes, then the coordinates of a gaze target fixation point are calculated and the eye gazing recognition result is obtained.

**Figure 22:**
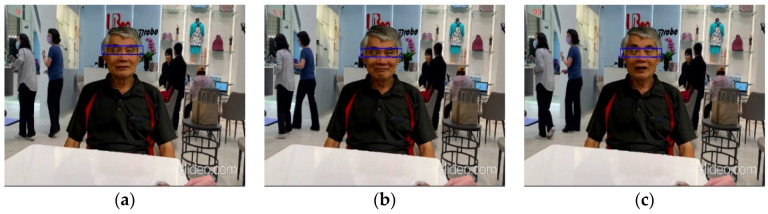
Representative eye gazing recognition results from the video of an elderly man: (**a**) Frame 9 (gazing to the left); (**b**) Frame 31 (gazing at the center); (**c**) Frame 99 (eyes closing).

To evaluate the recognition efficacy of the care robot for elderly people, we filmed many videos that took place in an indoor environment and verified the accuracy of the eye gazing recognition. The following example is the close-up video (length: 30 s) of an elderly man, and we randomly chose the last 100 frames to test. Figure 22 demonstrates some representative results of the eye gazing recognition from this video.

Table 5 is a confusion matrix of the statistics of the eye gazing recognition from the above example. Table 6 lists the evaluation data obtained from the confusion matrix, which reveals excellent recognition performance.

### 4.4. Chinese Speech Recognition Test

**Figure 23:**
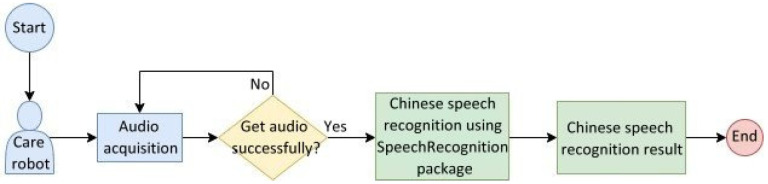
Flowchart of the Chinese speech recognition system embedded in care robots.

To simulate a Chinese speech recognition test scenario for the elderly, Figure 23 shows a flowchart of the Chinese speech recognition system designed by this study for use in care robots. The robot acquires the speech information of the elderly through its microphone and repeats the attempts until the acquisition is successful; if the speech information is obtained, the robot performs Chinese speech recognition. This functionality is realized by the SpeechRecognition package [22] that converts speech information to text and analyzes the meaning.

To evaluate whether the SpeechRecognition library used in the study is capable of Chinese speech recognition, we adopt the Common Voice dataset [23] to verify the accuracy of speech recognition. The Common Voice dataset records 67,491 voice files of Chinese sentences. The word error rate (WER) was used as the standard to verify the Common Voice dataset, which is depicted in Equation (6).

(6) $$Word\;error\;rate=\frac{I+D+S}{N}\times 100\%$$

where N represents the number of correct words in the reference sentence; I is the number of inserted unrecognized words when the recognized words are less than the reference; D is the number of words deleted when the recognized words are more than the reference, and S is the number of substituted words to match the reference. In short, the WER standard utilizes the Levenshtein distance; that is, the concept of the minimum editing distance, in order to compute the difference between the recognition result and correct reference.

In the Common Voice dataset, our SpeechRecognition package is able to achieve a WER of 13.21% which is considered a satisfactory result. We observed that the Common Voice dataset contains a certain percentage of erroneous data that increases the wrong results and increases WER; for example, microphone noise completely drowns out the human speech, or some phrases are spoken in Cantonese. Based on the experiments, it seems that the SpeechRecognition package is reliable and accurate in recognizing the Chinese speech of elderly people.

## 5. Conclusions and Further Implementation

After reviewing the literature and proposing strategies for improvement, we applied image and speech recognition techniques to accomplish four built-in functions developed for care robots currently. These functions are human pose recognition, human facial expression recognition, eye gazing recognition, and Chinese speech recognition described as follows.

### 5.1. Current Achievements

#### 5.1.1. Human Pose Recognition

We used a video camera to simulate the height and inclination angle of the eyes of a care robot to record elderly people’s posture images that are then used to train the human pose recognition model. The MediaPipe framework is used to detect the positions of the major human body joints in 3D space and convert them into a vector for detecting a fall event. Through the LSTM-based architecture of the elderly fall detection model, there are five kinds of human poses that can be recognized: walking, squatting, sitting, lying down, and falling.

#### 5.1.2. Human Facial Expression Recognition

Human face images captured by the camera equipped on the care robot were put into the convolutional neural network that mainly consisted of four convolutional layers for training. From this, the trained model can conduct recognition of elderly people’s facial expressions, including seven kinds: anger, disgust, fear, happiness, sadness, surprise, and neutral.

#### 5.1.3. Eye Gazing Recognition

For the human face image captured by the camera, landmarks were labeled to locate human eyes and calculate the coordinates of the gaze target fixation point via a formula deduced from geometry relationship between eyes and a target. The gazing recognition results include eyes closing, gazing to the left, the right, and at the center.

#### 5.1.4. Chinese Speech Recognition

The Chinese speech from elderly people was received by the microphone equipped on the care robot and converted into words and sentences. They were then segmented and passed through a CBOW model to predict the middle words using surrounding context words. This allows the key meanings of elderly people’s spoken phrases to be obtained. Based on the results of phrase prediction, text for specific key phrases is generated and then converted into human-like speech as output.

Although we have obtained preliminary results on the four functions built for care robots, there remains room for improvement. The following is the brief descriptions of future work for further implementation of human pose recognition, human facial expression recognition, eye gazing recognition, and Chinese speech recognition.

### 5.2. Future Work

#### 5.2.1. Human Pose Recognition

Because the deep learning model of human pose recognition is too large, it cannot be effectively used under the condition of system integration. Therefore, in future work, this study will continue to optimize the architecture of the human pose recognition model and strive to improve its accuracy.

#### 5.2.2. Human Facial Expression Recognition

At present, the deep learning model used in the facial expression recognition of this study already has complete functions. Therefore, we will focus on the integration of the system, try to reduce the misjudgment of facial expression image recognition, and do our best to improve the potential performance delay due to integration.

#### 5.2.3. Eye Gazing Recognition

In this study, traditional image recognition methods are used for eye gazing recognition. While they serve the purpose, there is still room for improvement. We will continue to perfect the techniques for eye gazing recognition and improve the system integration performance to avoid interference from executing the other three tasks.

#### 5.2.4. Chinese Speech Recognition

In terms of system integration, it was found that multi-threading interferes with Chinese speech recognition most frequently. We will continue to investigate how the care robot can engage in fluent speech interaction with elderly people in the case of multi-threading, and collect more scenario script examples for our Chinese speech database.

## Figures and Tables

**Table 1:** Confusion matrix for the pose detection of a falling elderly man.

	Prediction	Walking	Squatting	Sitting	Lying Down	Falling
Actual						
Walking		28	0	0	0	0
Squatting		0	24	2	0	2
Sitting		0	2	22	0	5
Lying down		0	0	0	25	6
Falling		1	5	8	11	27

**Table 2:** Evaluation data for the pose detection of a falling elderly man.

	Pose	Walking	Squatting	Sitting	Lying Down	Falling
Evaluation Metric						
Precision		96.5%	77.4%	68.8%	69.4%	67.5%
Recall		100%	85.7%	73.3%	78.1%	51.9%
Accuracy		74.1%	74.1%	74.1%	74.1%	74.1%
Harmonic mean		98.2%	81.3%	71.0%	73.5%	58.7%

**Table 3:** Confusion matrix of human facial expression recognition.

	Prediction	Anger	Disgust	Fear	Happiness	Sadness	Surprise	Neutral
Actual								
Anger		76	1	4	7	5	2	5
Disgust		0	83	4	3	5	3	2
Fear		0	0	97	0	2	1	0
Happiness		0	2	3	90	3	1	1
Sadness		0	3	4	2	91	0	0
Surprise		0	1	5	1	1	91	1
Neutral		0	1	3	1	5	3	87

**Table 4:** Evaluation data of human facial expression recognition.

	Expression	Anger	Disgust	Fear	Happiness	Sadness	Surprise	Neutral
Evaluation Metric								
Precision		99.5%	90.8%	80.9%	87.1%	80.8%	90.0%	90.6%
Recall		75.6%	83.2%	96.5%	89.9%	91.3%	91.4%	86.6%
Accuracy		87.8%	87.8%	87.8%	87.8%	87.8%	87.8%	87.8%
F1-score		85.9%	87.1%	88.0%	88.3%	85.7%	90.9%	88.8%

**Table 5:** Confusion matrix of the eye gazing recognition results of the elderly man.

	Prediction	Gazing to the Left	Gazing at the Center	Gazing to the Right	Eyes Closing
Actual					
Gazing to the left		0	0	0	0
Gazing at the center		0	24	0	0
Gazing to the right		0	8	65	0
Eyes closing		0	0	3	0

**Table 6:** Evaluation data of the eye gazing recognition results of the elderly man.

	Gaze State	Gazing to the Left	Gazing at the Center	Gazing to the Right	Eyes Closing
Evaluation Metric					
Precision		N/A	100.0%	89.0%	N/A
Recall		N/A	75.0%	95.5%	N/A
Accuracy		89.0%	89.0%	89.0%	89.0%
Harmonic mean		N/A	86.0%	92.0%	N/A

## Data Availability

Not applicable.
